# Care of Children's Teeth between the Ages of Six and Fifteen

**Published:** 1874-07

**Authors:** P. E. White

**Affiliations:** Davenport, Ia.


### ARTICLE II.

### *Care of Children's Teeth, between the Ages of Six and Fifteen.*

BY P. E. WHITE, DAVENPORT, IA.

Read before the Illinois State Dental Society.

We all recognize the vast increase in number of our "little patients" suffering from disease of the dental organs, and, in consequence, one of the practical questions of the hour is—how shall we care for them? I do not propose to enter into the *whys* and *wherefores* of the diseased conditions of the teeth, but commence where I find my patient, and explain my practice as they come to me daily. The first question that presents itself to me is,—*Should the deciduous teeth receive the same care and attention as the permanent?* I answer, YES. Our creator evidently gives the deciduous teeth as important functional duties to perform as the permanent; and when we glance at the process of nature, and find certain laws in force, with such accuracy that we can determine the time of the eruption of the several teeth, both deciduous and permanent, we certainly ought not to question the goodness of Him who has pronounced all things good, but must acknowledge that all His works were made for a wise purpose, and to perform their particular part in life.

In order then that the question may be answered satisfactorily, let us look at the duty of the temporary teeth; we find that they are erupted at an age, and as rapidly as the system requires more solid nutriment, continuing its work until the full development of the teeth has been obtained; in normal conditions we find incisors, cuspids and molars, properly adapted and in perfect articulation; they are then placed for mastication of food, subduing the solid elements, preparatory to being introduced into the stomach; giving space for the full development of the permanent teeth, and as the process continues and absorption takes place, it is even already contended by some that the elements contained in the deciduous teeth may be appropriated as nutriment in the formation of the permanent. The teeth give the child the use of speech, giving him advantages from the first, of learning right pronunciation, and when erupted at the proper age, oftentimes preventing the lisp which may be caused by a too free escape of sound. They also give a perfect contour of the face, producing symmetry and beauty of the features; in fact they are needed for the *perfect development* of the child.

The premature loss of the deciduous teeth, I think, occasions more injury to the dental organism than the loss of a permanent tooth after a full development of the maxillas. If then the temporary teeth are of so much importance, can there be but one answer, as to whether they should receive the greatest of care? Is there one among us to-day, but that would most strenuously oppose the unnecessary removal of a permanent tooth, however much it might be affected by disease, but on the contrary, make as earnest efforts in the opposite direction, saving the tooth for its proper function. Just such a stand do I take in regard to retaining the temporary dental organs intact, until by process of nature, the time shall have come for their removal, either naturally or by surgical means. I most certainly believe that the amount of injury being done to our young friends by neglect, through ignorance and wilfulness, is incalculable; and that every effort should be exercised, and all means taken for the preservation of their teeth, is one of the duties of the dentist; at no time in life do I consider that the teeth require the attention and care requisite for their future usefulness, as between the ages of six and fifteen; the great changes that transpire in the dental economy within that time, is simply wonderful, such as no student of nature can witness without exclaiming as did David, "I am fearfully and wonderfully made."

The design of nature was evidently perfect, yet how do we find it in our practice? In this degenerate age, when people do not live over righteously, we find the teeth of the young becoming carious at a fearful rate; and unless we bestir ourselves and give the people a knowledge in regard to the subject, as is our duty, if I mistake not, there will be more mechanical dentistry to perform for the coming generation than has ever been done within the same time previously.

Our young people are coming to us daily for relief, already suffering with painful teeth, from vitiated conditions of the secretions of the mouth, tartar in all its various forms and chemical combinations, inflamed and ulcerated gums, oftentimes attended with a discharge of purulent matter from around the necks of the teeth—morbid state of the absorbents, these and kindred lesions of first dentition should receive judicious management; above all no tooth should be removed only as a *dernier resort*, but should, if diseased, be treated and filled, that it may occupy in usefulness its allotted position.

The premature extraction of the teeth brings me to the point of *irregularity*. Within the past year I have examined closely mouths of many children and adults, frequently taking casts of such as were particularly interesting in regard to this subject ; my observations thus far most certainly lead me to the conclusion that *improper treatment of the temporary teeth*, is as a *rule*, the *primary cause of irregularity of the permanent.*

A case in point:—I have in my possession, a cast taken from the mouth of a young lady aged nineteen, which exhibits the centrals overlapped, the laterals turned one quarter round, with their palatine surfaces pressing hard against the distal surfaces of the centrals—the right cuspid inside the palatine arch, while the left cuspid is outside, lying in almost a horizontal position, pointing toward the mesial line; the first bicuspids are wedged closely to the laterals, occupying the true position of the cuspids, while the molars are in their natural position. Learning the history of the case, I found that at the age of six, the centrals had been erupted, and that the laterals were forcing their way, causing considerable pressure upon the deciduous teeth; applying to a dentist for advice and treatment, he extracted the temporary laterals and *cuspids*, hoping thereby to give plenty of space for the eruption of the coming laterals. He succeeded admirably, as the foregoing description, and a glance at the model will show. The great mistake made, was in removing the cuspids, for had they been allowed to remain until the age or circumstances warranted their extraction, or until sufficient expansion of the maxillas had occurred, I am convinced that no such fearful result would have followed. [Note :—I would state that this case was most successfully treated by the removal of the second bicuspid on the right side, and the first bicuspid on the left, when by the aid of proper plates and appliances, the teeth assumed a beautiful and symmetrical appearance.]

Says Prof. Garreston :—" If for one moment we refer to certain physiological relations existing between the first and second dentures, we may find that it is within our power to prevent the many ills that follow so frequently in this train, and simply by little or, more commonly, nothing. The deciduous dental arch is filled completely by its ten teeth. The permanent set is to comprise in number, six·teen, and each tooth certainly quite as large again as its predecessor. This increase in number and size of the teeth, it is evident must be provided for in an enlargement of the alveolar arch. This provision is always attempted by nature in the process described by the physiologist as elongatory. * * * *In proportion to the number of deciduous teeth removed prematurely, will be the curtailment in size of that arch, at least of its alveolar face.*"

Much, however, must depend upon the skillful judgment of the operator in directing second dentition, and in order that success for the futnre may be assured, rather than to be hasty in our conclusions, let us wait till we have satisfied ourselves as to what results we are aiming to accomplish, diagnosing the case with care, and never thinking it lowering our professional dignity, by seeking the advice of a brother who perhaps may know more than ourselves.

The above may suffice, perhaps, to introduce the subject of prevention of irregularity of the permanent teeth, and as to how we shall proceed after the irregularity has already been gained, is a question with which we are all familiar ; here ingenuity displays itself in the many various modes of treatment, appliances, &c., and where perfection can be gained, often restores mal-formed dentures to usefulness and beauty. I would raise just one objection in the extracting for treatment of irregularity, which to me seems of much importance; of course, judgment must be used in every case presented, but to me, the practice of some, who extract the first permanent or six-year molar, is exceedingly erroneous, and detrimental to the future formation of the maxillas. I have observed during the past, mouths where the molars have been extracted, where *proper articulation* has been wholly destroyed ; in almost every case there has been space sufficient for a bicuspid, that there is no probability of ever being filled, and with the second molars pitching forward at a considerable angle, with its antagonizing tooth merely touching on a small portion of it, if at all ; with such cases, had the second bicuspid been removed, there would have been plenty of room to have regulated successfully all the teeth anterior to them, while the antagonism would have remained perfect.

Now as to the general care of the teeth, during the ages I have selected. I find no better treatment than to follow strictly the common laws of hygiene ; particularly do I urge upon parents the very great importance, in order that their children may have sound, healthy teeth, free from caries and pain, that there should be perfect cleanliness of the mouth and body, proper clothing, good substantial nutritious food, plenty of free, healthy exercise in the open air, and—rest. Whenever I examine the teeth of a child, I look closely to the condition of the six-year molars, so often mistaken by parents as being the temporary teeth ; and should the child's mother be present, I explain to her by charts and models, the great necessity that those teeth should be preserved, while the thanks that I have so often received for so doing have been many, paying me better than any pecuniary consideration.

Recommend and urge to parents that the teeth of their children should be examined often, that they should, when found carious and diseased, be properly treated and filled, never omitting, when the parents or children are anxious to receive instruction on this point, to explain, as far as possible, in a general way, the process of first and second dentition, and the many diseases often attending them. I cannot better close this paper, than to show how diet and a regularity in life may affect the dental organism, and in fact the whole body.

I have lately been allowed to visit and examine the mouths of the children connected with the Orphan's Home, located near our city, through the courtesy of the superintendent, Mr. S. W. Pierce. Thinking that the natural and regular course of living among the children must exert a marked effect upon the teeth, induced me to make the examination, and the result was far beyond my most sanguine expectations. Here let me give a few statistics. The Home has been organized nearly nine years, during this time, over 1,100 children have been enrolled, largest number at any one time 585; there are now present 180 (the smallest number ever at the Home) boys and girls, whose ages are from five to sixteen years. Of the remarkable health of the institution, I need state but one fact, viz: there has been but *one death* among the children since August, 1869. This is an extraordinary showing, and it would be well for all who are interested in the health of our children, and even of ourselves, to inquire into the reasons why, connected with it. 1st.—The Home is located on a charming spot, grounds elevated, and where plenty of pure fresh air can be obtained, and the sun has free access to all parts of the grounds. 2d.—Strict attention is paid to proper exercise, both of mind and body, and thorough cleanliness of the homes and the inmates enforced. 3rd.—The children are regular and methodical in all their ways, whether in school, at work or play, eating or retiring. 4th—Proper attention is required in regard to the diet, and herein, it seems to me, lies one of the great secrets of success, and one, although we know its truths, yet are so loth to practice. It may not be amiss for me to present to your notice, a list of the articles of diet which is prescribed for the children.

BREAKFAST.

Sunday—Baked beans, brown and white bread and butter.

Monday—Boiled milk, bread and syrup.
Tuesday—Meat stew, bread and syrup.
Wednesday—Baked beans, bread and butter.
Thursday—Boiled milk, bread and syrup.
Friday—Boiled potatoes, bread and butter.
Saturday—Meat stew, bread and syrup.

DINNER.

Sunday—Cold meat, pickles, bread, butter, cheese, apples and pie.

Monday—Vegetable soup, cold slaw, corn bread, butter and apples.

Tuesday—Potato soup, pickles, rice and syrup.
Wednesday—Hash, onions, bread, butter and apples.
Thursday—Noodle soup, baked potatoes, bread and butter
Friday—Bean soup, rice, or hominy, apples and syrup.
Saturday—Hash, onions, bread and butter,

SUPPER.

Sunday—Luncheon of cake and fruit.
Monday—Bread and butter or syrup.
Tuesday—Bread and milk and gingerbread.
Wednesday—Mush and milk.
Thursday—Corn bread and butter or syrup.
Friday—Rusk, bread and milk, and gingerbread.
Saturday—Bread and milk.

Other fruits and vegetables in their season. Drinks—milk and water.

Upon a diet like this, for seven or eight years, and with the regulations required, our theory would be that the dental organism would, or ought to be perfect. Now as to facts,—to my great surprise, knowing the average condition of the teeth of children in the city, in the large number of oral cavities examined, I did not find but three or four temporary teeth at all carious. I found the process of first and second dentition taking about its ordinary course as to time of eruption, yet, if anything, second dentition was retarded somewhat, owing to the extremely firm and solid material contained in the temporary teeth. In my further observation, I found but two or three of the permanent teeth at all decayed, excepting in one little girl of a scrofulous or syphilitic diathesis, who had been in the home only two years when the six-year molars were carious to quite an extent, the crowns to three of the teeth being entirely gone.

The cuspids, bicuspids and second molars of those examined, and especially of those who had been in the Home for six or seven years, were most beautiful, exhibiting that strong, healthy texture in appearance, that indicates perfect development of the tooth structure.

I learned that the children seldom, if ever, used a brush or pick upon the teeth, and yet salivary calculus was not present to any great extent, except in a few who were suffering from hereditary or constitutional disease; but this —uncleanliness of the mouth and teeth—was the only thing to mar the beauty of the whole; otherwise, picture to yourselves how teeth, and in fact the children themselves should look, living under such a regimen, and you have it full exemplified in the children now at the Orphan's Home. Much credit must be due the superintendent, matron, and assistants, for the thorough manner in which the common hygienic laws are observed, in the valuable institution over which they have charge.

The lessons that I have learned myself, from my visit to the Home, have been many and valuable; and perhaps the greatest is,—if I would wish to live long, and free from the many ills and sufferings of life, proper and strict attention should be given to exercise, cleanliness, diet and rest.

